# Network-based characterization of brain functional connectivity in Zen practitioners

**DOI:** 10.3389/fpsyg.2015.00603

**Published:** 2015-05-12

**Authors:** Phebe B. Kemmer, Ying Guo, Yikai Wang, Giuseppe Pagnoni

**Affiliations:** ^1^Department of Biostatistics and Bioinformatics, The Rollins School of Public Health, Emory UniversityAtlanta, GA, USA; ^2^Department of Biomedical, Metabolic and Neural Sciences, University of Modena and Reggio EmiliaModena, Italy

**Keywords:** meditation, fMRI, functional connectivity, sustained attention, network analysis

## Abstract

In the last decade, a number of neuroimaging studies have investigated the neurophysiological effects associated with contemplative practices. Meditation-related changes in resting state functional connectivity (rsFC) have been previously reported, particularly in the default mode network, frontoparietal attentional circuits, saliency-related regions, and primary sensory cortices. We collected functional magnetic resonance imaging data from a sample of 12 experienced Zen meditators and 12 meditation-naïve matched controls during a basic attention-to-breathing protocol, together with behavioral performance outside the scanner on a set of computerized neuropsychological tests. We adopted a network system of 209 nodes, classified into nine functional modules, and a multi-stage approach to identify rsFC differences in meditators and controls. Between-group comparisons of modulewise FC, summarized by the first principal component of the relevant set of edges, revealed important connections of frontoparietal circuits with early visual and executive control areas. We also identified several group differences in positive and negative edgewise FC, often involving the visual, or frontoparietal regions. Multivariate pattern analysis of modulewise FC, using support vector machine (SVM), classified meditators, and controls with 79% accuracy and selected 10 modulewise connections that were jointly prominent in distinguishing meditators and controls; a similar SVM procedure based on the subjects’ scores on the neuropsychological battery yielded a slightly weaker accuracy (75%). Finally, we observed a good correlation between the across-subject variation in strength of modulewise connections among frontoparietal, executive, and visual circuits, on the one hand, and in the performance on a rapid visual information processing test of sustained attention, on the other. Taken together, these findings highlight the usefulness of employing network analysis techniques in investigating the neural correlates of contemplative practices.

## Introduction

In recent years, brain-wise functional connectivity analyses have become an increasingly important tool for understanding normal brain function as well as its alterations across specific subpopulations (Lowe, 2012). In particular, the investigation of intrinsic connectivity networks by resting state functional magnetic resonance imaging (fMRI) has proven capable of revealing fundamental elements of human brain architecture and organization. Resting state functional connectivity (rsFC) analyzes the temporal correlations of the spontaneous BOLD signal fluctuations across the brain in the absence of any experimental task, a reflection of the neural activity intrinsically generated by the brain (Fransson, 2005; Fox and Raichle, 2007). Since Biswal et al. (1995) first noted the preservation of the functional connectivity structure of the sensorimotor cortical network during rest, several other resting state networks (RSNs) have been consistently identified in the human brain (Smith et al., 2009; Zuo et al., 2010; Allen et al., 2011; Laird et al., 2011), with a particular emphasis on the so-called default mode network (DMN; Raichle et al., 2001; Greicius et al., 2004). Furthermore, significant differences in intrinsic connectivity networks across clinical and demographic subpopulations have also been reported (Bassett et al., 2008; Dosenbach et al., 2010; Satterthwaite et al., 2014).

Brain imaging has been employed quite extensively in the last decade to explore the potential neural changes associated with contemplative practices. In particular, functional connectivity alterations in DMN areas, frontoparietal attentional circuits, saliency-related regions, as well as in primary sensory cortices, have been observed in experienced meditators compared to meditation-naïve controls (Farb et al., 2007, 2013; Brewer et al., 2011; Jang et al., 2011; Josipovic et al., 2011; Kilpatrick et al., 2011; Froeliger et al., 2012; Hasenkamp and Barsalou, 2012; Taylor et al., 2013; Garrison et al., 2014). We have also previously investigated the neural correlates of conceptual processing associated with the practice of Zen meditation using fMRI and a lexical decision task (Guo and Pagnoni, 2008; Pagnoni et al., 2008; Guo, 2011; Guo and Tang, 2013); in the same sample of volunteers (12 practitioners of Zen meditation and 12 matched control subjects), we have additionally reported on the functional connectivity and temporal properties of the BOLD signal from the posterior cingulate cortex, the main node of the DMN, during a meditative attention-to-breathing condition (Pagnoni, 2012).

In the present work, we further examined the fMRI data collected during the attention-to-breathing condition in the cohort of subjects described in Pagnoni (2012), aiming to detect differences in rsFC between meditators and controls across the whole brain using a network approach. In order to obtain a fine-grained parcellation of the whole cerebral cortex, we adopted the 264-node system proposed by Power et al. (2011). We also assigned the nodes to a set of nine functional modules that were consistently identified as RSNs in larger populations (Smith et al., 2009). Network connectivity differences between meditators and controls were assessed using modulewise and edgewise comparisons of network connections and multivariate pattern analyses via a support vector machine (SVM) classifier. The multivariate pattern analyses aimed to find a hyperplane based on the high-dimensional pattern of brain connectivity measures able to separate meditators from control subjects. Finally, we examined the association between selected brain connectivity measures and individual scores on a rapid visual information processing (RVIP) test of sustained attention and working memory.

## Materials and Methods

### Subjects

Twelve Zen meditators with more than 3 years of daily practice (MEDT) were recruited from the local community and meditation centers, along with 12 control subjects (CTRL) who never practiced meditation. All the volunteers in the meditators group had more than 3 years of daily practice of zazen (Zen objectless meditation) under the guidance of a certified teacher (mean = 8.7 years, SD = 6.5 years, minimum = 3 years, maximum = 20 years). Almost all of them (11/12) were practicing within the Zen Soto tradition, but a few of them also had some experience with different contemplative practice styles (Zen Rinzai 3/12, Tibetan 2/12, Vipassana 2/12); three meditators were ordained Soto monks. The meditator and control groups were matched for gender (MEDT: 10 M, CTRL: 9 M), age (mean ± SD: MEDT, 37.3 ± 7.2 years; CTRL, 35.3 ± 5.9 years; 2-tailed, 2-sample *t*-test: *p* = 0.45), and education level (mean ± SD: MEDT, 17.8 ± 2.5 years; CTRL, 17.6 ± 1.6 years; *p* = 0.85). All participants were native speakers of English and right-handed, except one meditator who was ambidextrous. Subjects gave written informed consent for a protocol approved by the Emory University Institutional Review Board.

### Neuropsychological Computerized Testing

Approximately 1 week before the MRI scanning session, every volunteer completed a selected subset of the CANTAB computerized neuropsychological battery (Sahakian and Owen, 1992). The CANTAB tests included: (1) a task assessing sustained attention (RVIP) in terms of sensitivity to the target (*A’*, a non-parametric analog of *d’* from signal detection theory) and response time; (2) a test of simple reaction time (RTI simple) to a visual stimulus appearing in a fixed location on the computer screen, in terms of reaction time proper (time to raise the finger from the resting pad) and movement time (time to reach the target on the touch screen with the finger); (3) a complex reaction time task (RTI five choice), similar to the previous one, but where the visual target appeared randomly in one of five screen locations; (4) a test of visuospatial working memory capacity (Spatial Span, SSP), assessed in terms of span length (number of remembered items); (5) a test of rule learning and rule-switching [Intra–Extra Dimensional Set Shift (IED)] with two sets of rules (“intradimensional” and “extradimensional”) that assess cognitive flexibility in terms of the number of completed stages of progressive difficulty and number of errors for intradimensional and extradimensional rule learning. The reader can find a demo and a longer description of the tasks at http://www.cambridgecognition.com/academic/cantabsuite.

### MRI Acquisition and Preprocessing

A T1-weighted high-resolution anatomical image (MPRAGE, 176 sagittal slices, voxel size: 1 mm isotropic) and a single series of resting state functional images (echo-planar, 200 volumes, 35 axial slices, voxel size: 3 mm isotropic, TR = 2.35 s, TE = 30 ms) were acquired with a 3.0 Tesla Siemens Magnetom Trio scanner. Participants were instructed to keep their eyes open and pay attention to their breathing throughout the full length of the run (∼8 min), and to calmly return their attention to breathing every time they found themselves distracted by thoughts, memories, or physical sensations. A fixation cross was kept on the MRI display screen to help concentration and minimize eye movement. The functional volumes were corrected for slice acquisition timing differences and head motion. The anatomical image was first registered to the mean of the corrected functional images and then spatially warped to the MNI standard brain space by using the segmentation routine of SPM5 (http://fil.ion.ucl.ac.uk/spm/software/spm5). The estimated warping parameters were subsequently applied to the functional images, which were finally smoothed with an 8 mm isotropic Gaussian kernel. Low-frequency signal drifts were removed from the time series by regressing out a Legendre polynomial of order two.

### Network Construction and Functional Module Parcellation

We adopted the 264-node cortical parcellation system defined by Power et al. (2011), where each node is a 10 mm diameter sphere in MNI space representing a putative functional area. Such a parcellation was determined using a combination of meta-analysis of task-based fMRI studies and rsFC mapping techniques; it constitutes a finer grid compared to the Automated Anatomical Labeling (AAL) set (Tzourio-Mazoyer et al., 2002), but is not as granular as a collection of single voxels. It thus represents an appealing choice, in that it provides a good balance between spatial localization and dimension reduction (Fornito et al., 2010; Power et al., 2011).

**FIGURE 1:**
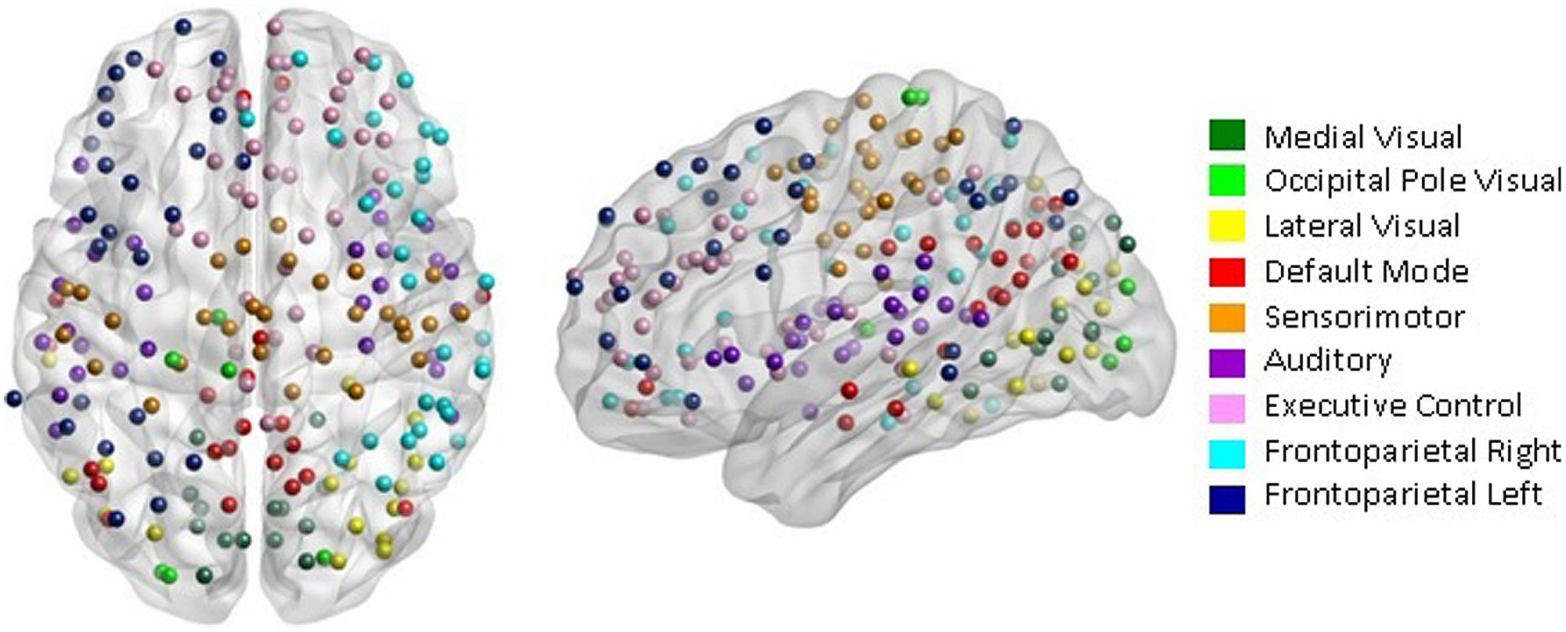
**Parcellation scheme and network assignment.** The 209 nodes used in our network analysis are adapted from the 264-node parcellation system defined by Power et al. (2011). Each node is a 10 mm diameter sphere in MNI space representing a putative functional area, and is color-coded to indicate its module membership. Functional modules are defined by the 10 primary resting state networks (RSNs) described in Smith et al. (2009).

**FIGURE 2:**
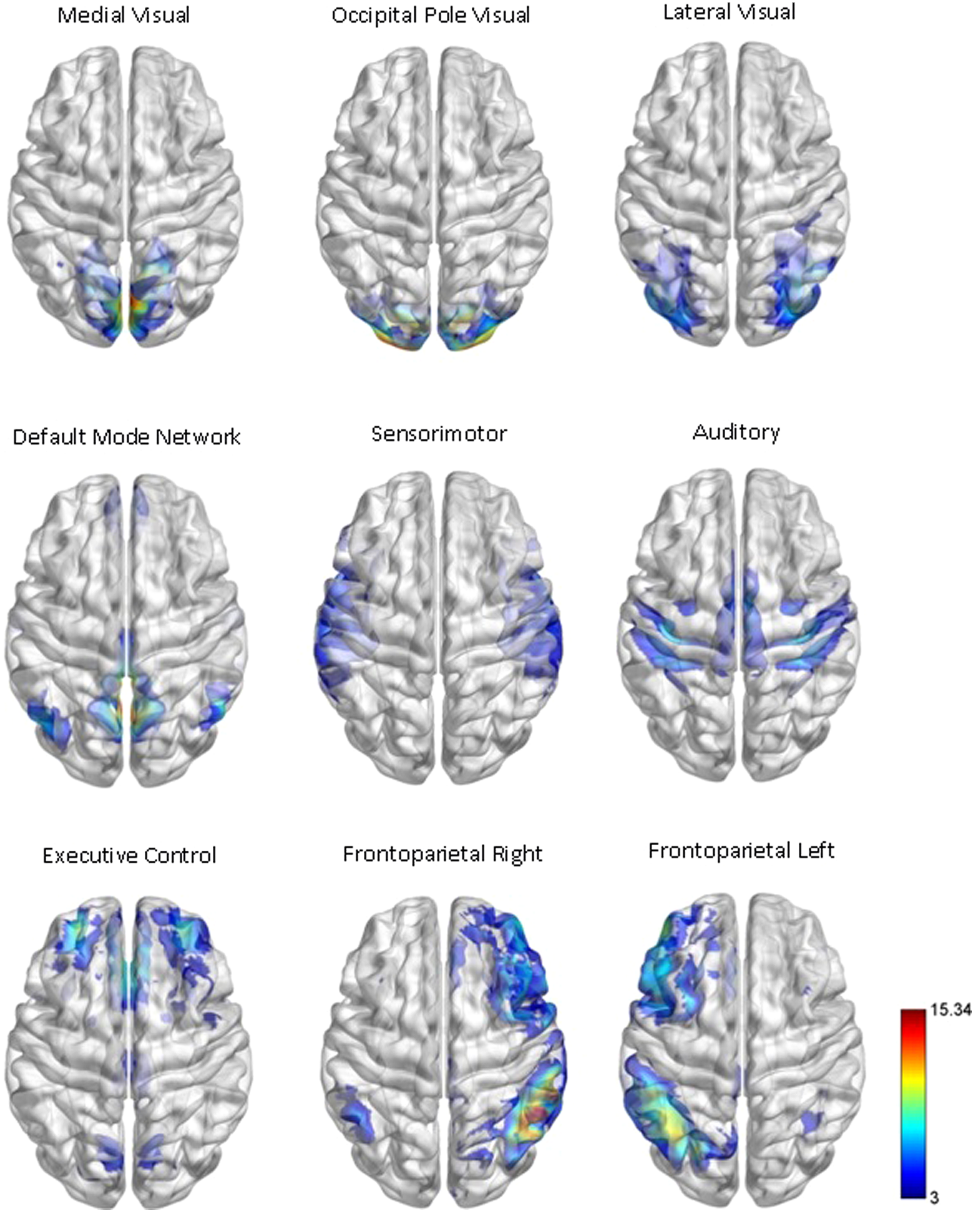
**Functional module maps.** The functional module *z*-score maps (thresholded at *z* > 3) defined by the 10 primary RSNs described in Smith et al. (2009). To categorize nodes by module membership, we find the RSN map with the largest *z*-score in the location of the node, above a certain threshold (*z* > 3). The module corresponding to the cerebellum is not shown or used in our analysis, as it contained only one node in gray matter.

Our network analysis considered 238 of the 264 nodes that were within the boundary of the data gray matter mask. We assigned these nodes to nine functional networks or “modules” that correspond to the major RSNs described by Smith et al. (2009). The RSN maps, determined by ICA decomposition of a large database of activation studies (BrainMap) and resting state fMRI data, are circuits whose BOLD activity is temporally coherent during both task activity and at rest. The functional modules include a medial visual network (“Med Vis,” 14 nodes), an occipital pole visual network (“OP Vis,” six nodes), a lateral visual network (“Lat Vis,” 16 nodes), the “DMN,” 21 nodes, the cerebellum (one node), a sensorimotor network (“SM,” 29 nodes), an auditory network (“Aud,” 29 nodes), an executive control network (“EC,” 38 nodes), and a right and left frontoparietal network (“FPR” and “FPL,” 30 and 26 nodes, respectively). To determine the module membership at each node, we identified the RSN *z-*statistic map with the largest value in the location of the node, above a chosen threshold (*z* > 3). Twenty nine of the 238 nodes in gray matter were not strongly associated with any RSN map, and were therefore not included. Also, since only one node was contained in the cerebellum, the latter module and corresponding node were discarded as well. A visualization of the remaining 209 nodes that are used in the subsequent network analysis, classified by functional module, is shown in **Figure 1**. A map of the functional modules is also displayed in **Figure 2**. The parcellation of nodes into functional modules allows examination of within- and between-module connectivity. All brain visualizations were created using BrainNet Viewer (Xia et al., 2013).

### Graph Construction

To construct the network connectivity matrix, we extracted the time series from each node with the following steps. First, the time series at each voxel was detrended by regressing out a Legendre polynomial of second order, demeaned, and whitened. We then performed singular value decomposition (SVD) on the time series for all the voxels in each node to extract the representative time series within that node. A 209 × 209 symmetric connectivity matrix was defined for each subject by calculating Pearson correlations between the summary time series extracted from each node. To avoid the issue of arbitrary thresholding, our network analyses were conducted on fully connected graphs with both positive and negative weights. All graphical network visualizations were created using the igraph package in R (Csardi and Nepusz, 2006).

### Group Differences in Modulewise Functional Connectivity

In order to summarize the 21,736 unique edges in the 209-node network by the nine functional modules reported in Smith et al. (2009), we first performed a dimension reduction on the original subject-specific connectivity matrices. More in detail, we divided the upper-triangle of the 209 × 209 edgewise connectivity matrix into 45 modulewise blocks representing the nine within- plus the 36 between-module connectivity strengths. For each modulewise block *i* (*i* = 1,…,45; each containing m_i_ edges), we concatenated the block’s m_i_ edgewise connection strengths into a vector, then formed a (m_i_ × 24) block-specific connectivity matrix by stacking these vectors side by side across all 24 subjects. After removing the mean from each row, we performed a SVD on this matrix and extracted the first left singular vector (m_i_ × 1) and the first right singular vector (1 × 24). The first left singular vector represents the first principal component direction of block *i*’s connectivity pattern (i.e., one value for each edge in block, representative of all subjects), and the first right singular vector corresponds to the subject-specific coordinates when projecting each subject’s connectivity pattern on to the first principal component (i.e., one value per subject for each block *i,* representative of all the edges in the block). The set of right singular vectors, or principal component coordinates, can be seen as a compact representation of subjects’ module-to-module connectivity patterns, reducing the original 24 subject-specific edgewise connectivity matrices (209 × 209) to 24 subject-specific modulewise principal component coordinate matrices (9 × 9). For each of the 45 unique modulewise blocks, we calculated the standardized mean difference (Cohen’s *d*) in the modulewise principal component coordinates, and used an effect size threshold of 0.5 to identify group differences with at least a medium effect size (Cohen, 1988); for completeness, and as an exploratory inferential assessment, we also performed a non-parametric Wilcoxon rank sum test comparing meditators and controls on the principal component coordinates from each module-module block, reporting the associated uncorrected *p*-values. Finally, as we will describe in Section “Group Differences Based on Multivariate Pattern Analysis,” the modulewise principal component coordinates were also used as input to a multivariate pattern analysis aimed at identifying an overall pattern of module-to-module connectivity discriminating between meditators and controls.

Of note, one consequence of summarizing the original 209 × 209 edgewise connectivity matrices into the SVD-based compact 9 × 9 modulewise connectivity matrices is that these compact matrices no longer directly provide information on the predominant sign of the connectivity strength in each of the module-to-module block. In order to recover such information (i.e., whether the BOLD signals from two modules were on average positively or negatively correlated), we also computed the mean connectivity strength within each module-to-module block by averaging across all the edgewise correlations in the block (see **Figure 4**). This allowed us to interpret and display between-group differences in the SVD-based compact connectivity results.

### Inspection of Edgewise Connectivity and Consistency of Group Effects within each Module–Module Block

The group differences in functional connectivity observed in the modulewise (SVD-based) analysis were further inspected at the edgewise level and checked for within module-module block consistency with the following approach. For each of the 21,736 unique edges in the 209 × 209 symmetric connectivity matrix, we calculated the standardized group difference at each edge using Cohen’s *d*, and selected those edges with an effect size magnitude of at least 0.5, representing a medium or larger difference (Cohen, 1988) between meditators and controls. We then grouped the selected edges into the nine within- and 36 between-module connection blocks described above and, for each module block, we assessed the dominant direction of the group difference at the edge-level (i.e., MEDT>CTRL or CTRL>MEDT) by calculating a consistency metric. This metric, based on the well-known McNemar test for consistency between two outcomes, was defined as (N_m__>__c_ – N_c__>__m_)/(N_m__>__c_ + N_c__>__m_), where N_m__>__c_ and N_c__>__m_ are the number of supra-threshold edges in the module-module block for the MEDT>CTRL or CTRL>MEDT effects, respectively. Thus, a value of 0 for the above metric indicates a complete lack of edgewise consistency in the direction of the MEDT–CTRL effect within a module-module block, while a value of 1 (or -1) represents a full edgewise consistency. In order to screen out module-module blocks with low block-consistency for the group effect, we set a threshold for the consistency metric of 0.33, corresponding to an effect where there are at least twice as many edges in a block showing a group effect in one direction compared to the other. This procedure allowed us to identify module-module blocks with consistently stronger connections either in meditators or controls. In order to avoid drawing conclusions based on just a few edges, we only calculated the consistency metric on module-module blocks where at least 5% of the total edges met the |*d*| > 0.5 threshold. We separately examined the thresholded edges with positive and negative average connectivity in both groups.

### Group Differences Based on Multivariate Pattern Analysis

In addition to the modulewise and edgewise univariate analyses, we also performed a multivariate analysis aimed at identifying an overall pattern of module-to-module connectivity discriminating between meditators and controls. The information from all 45 module-to-module SVD-derived summary measures was thus used jointly as input to a SVM classifier to predict group membership for each volunteer, using the LIBSVM package (Chang and Lin, 2011).

The SVM model produced three important outcomes. First, we evaluated the accuracy of the SVM classifier using a fourfold cross-validation procedure, in which the multivariate model was trained on 75% of the data, and then tested on the remaining 25% of the data. The results based on the cross-validation procedure reflect how accurately we can identify meditators on the basis of their brain connectivity features. Second, we obtained a vector of weights of the 45 modulewise connectivity features on the multivariate classifier, which represent the relative importance of these features in discriminating meditators and controls. Third, for each subject, the SVM yielded a dimensional classification score, a continuous predictor that reflects the confidence with which a volunteer was classified as a meditator or control.

We also performed an additional SVM analysis to classify meditators and controls, based this time on the outcome measures of the five CANTAB tests described in Section “Neuropsychological Computerized Testing.” The accuracy rate of the CANTAB-based multivariate classifier was evaluated using the fourfold cross-validation procedure described previously, and compared to the accuracy rate from the modulewise connectivity-based SVM. From the SVM, we derived the CANTAB-based dimensional classification score, which reflects the degree to which a subject classified as a meditator or control, based on the subject’s neuropsychological performance. Finally, we tested whether the brain-connectivity-based dimensional scores and the CANTAB-based dimensional scores were significantly correlated; this analysis tested whether the brain connectivity patterns that differentiated meditators from controls were significantly associated with the neuropsychological profile that best discriminated meditators and controls.

Lastly, we conducted a third SVM analysis using as input the joint set of the SVD-based modulewise connectivity measures *plus* the CANTAB scores, and compared its classification accuracy to that of the SVM analyses based on modulewise connectivity or CANTAB scores alone.

### Association between Brain Connectivity Measures and Attentional Performance

As the sustained attention scores from the RVIP test have been previously shown to be sensitive to differential effects in meditators and controls (Pagnoni and Cekic, 2007; Pagnoni, 2012), we examined the correlation between each of the 45 modulewise first principal component coordinate values and the RVIP test scores, across all subjects (CTRL + MEDT). The purpose of this analysis was to identify a subset of the brain functional connectivity structure that was specifically associated with the capacity for sustained attention, a cognitive aspect generally assumed to be affected by meditative practice.

## Results

### Group Differences in Modulewise Functional Connectivity

**FIGURE 3:**
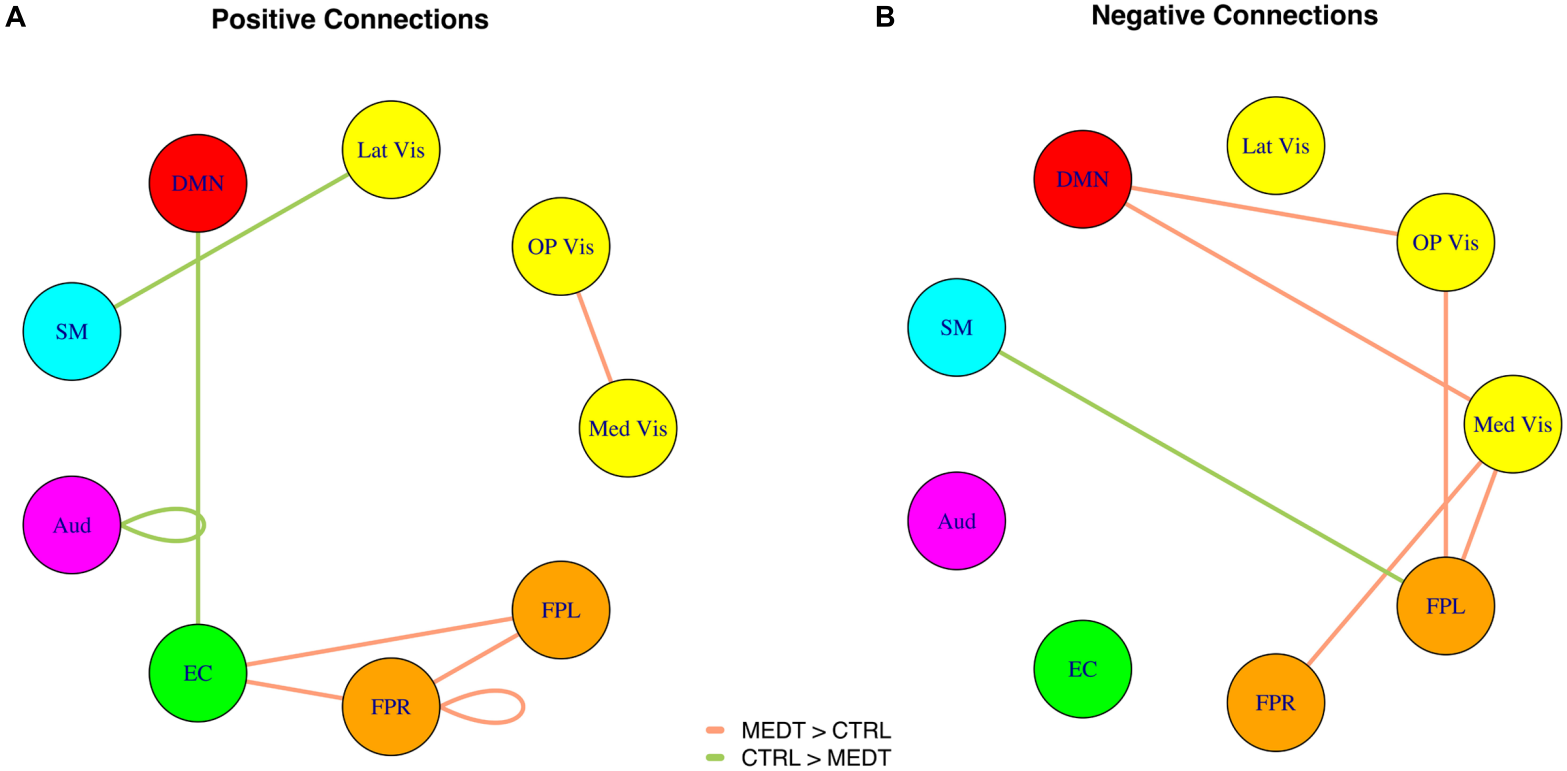
**(A)** Positive and **(B)** negative connections with at least a medium standardized mean difference (Cohen’s | *d*| > 0.5) between meditators and controls, as per the SVD-based modulewise analysis.

Meditators and controls were compared on the subject-specific modulewise principal component coordinates extracted from the SVD analysis, using Cohen’s *d* as a measure of effect size for each of the 45 module-module blocks. Among them, we identified 14 module-module connections with at least a medium-sized mean difference (i.e., Cohen’s *d* magnitude of at least 0.5) between meditators and controls. Module pairs with positive and negative average connectivity (as assessed by the original full edgewise correlation matrices, see Group Differences in Modulewise Functional Connectivity) for both groups are reported separately (**Tables 1A,B**), along with uncorrected *p*-values from the rank sum test. The modulewise results shown in this table are also displayed visually in **Figure 3**.

**Table 1A:** Group differences in positive modulewise functional connectivity.

Module-module connection	Standardized mean difference, *d*	Direction of difference	*p*-value (uncorrected)
Med Vis – OP Vis	0.77	MEDT>CTRL	0.126
Lat Vis – SM	-0.57	CTRL>MEDT	0.312
DMN – EC	-0.67	CTRL>MEDT	0.069
Aud – Aud	-0.53	CTRL>MEDT	0.403
EC – FPR	0.89	MEDT>CTRL	0.026
EC – FPL	0.57	MEDT>CTRL	0.046
FPR – FPR	0.71	MEDT>CTRL	0.157
FPR – FPL	0.56	MEDT>CTRL	0.141

Meditators had, on average, higher positive connections compared to controls for the Med Vis–OP Vis, EC–FPR, EC–FPL, FPR–FPR, and FPR–FPL module pairs. On the other hand, controls had, on average, higher positive connections for the Lat Vis–SM, DMN–EC, and Aud–Aud module pairs (**Figure 3A**).

**Table 1B:** Group differences in negative modulewise functional connectivity.

Module-module connection	Standardized mean difference, *d*	Direction of difference	*p*-value (uncorrected)
Med Vis – DMN	-0.54	MEDT->CTRL-	0.175
Med Vis – FPR	-0.80	MEDT->CTRL-	0.046
Med Vis – FPL	-0.78	MEDT->CTRL-	0.046
OP Vis – DMN	0.79	MEDT->CTRL-	0.069
OP Vis – FPL	-0.77	MEDT->CTRL-	0.069
SM – FPL	0.79	CTRL->MEDT-	0.100

Meditators had, on average, larger negative connection values for the Med Vis–DMN, Med Vis—FPR, Med Vis–FPL, OP Vis–DMN, and OP Vis–FPL module pairs, compared to controls; controls had higher negative connections compared to meditators for the SM–FPL module only (**Figure 3B**).

### Inspection of Edgewise Connectivity and Consistency of Group Effects within each Module–Module Block

**FIGURE 4:**
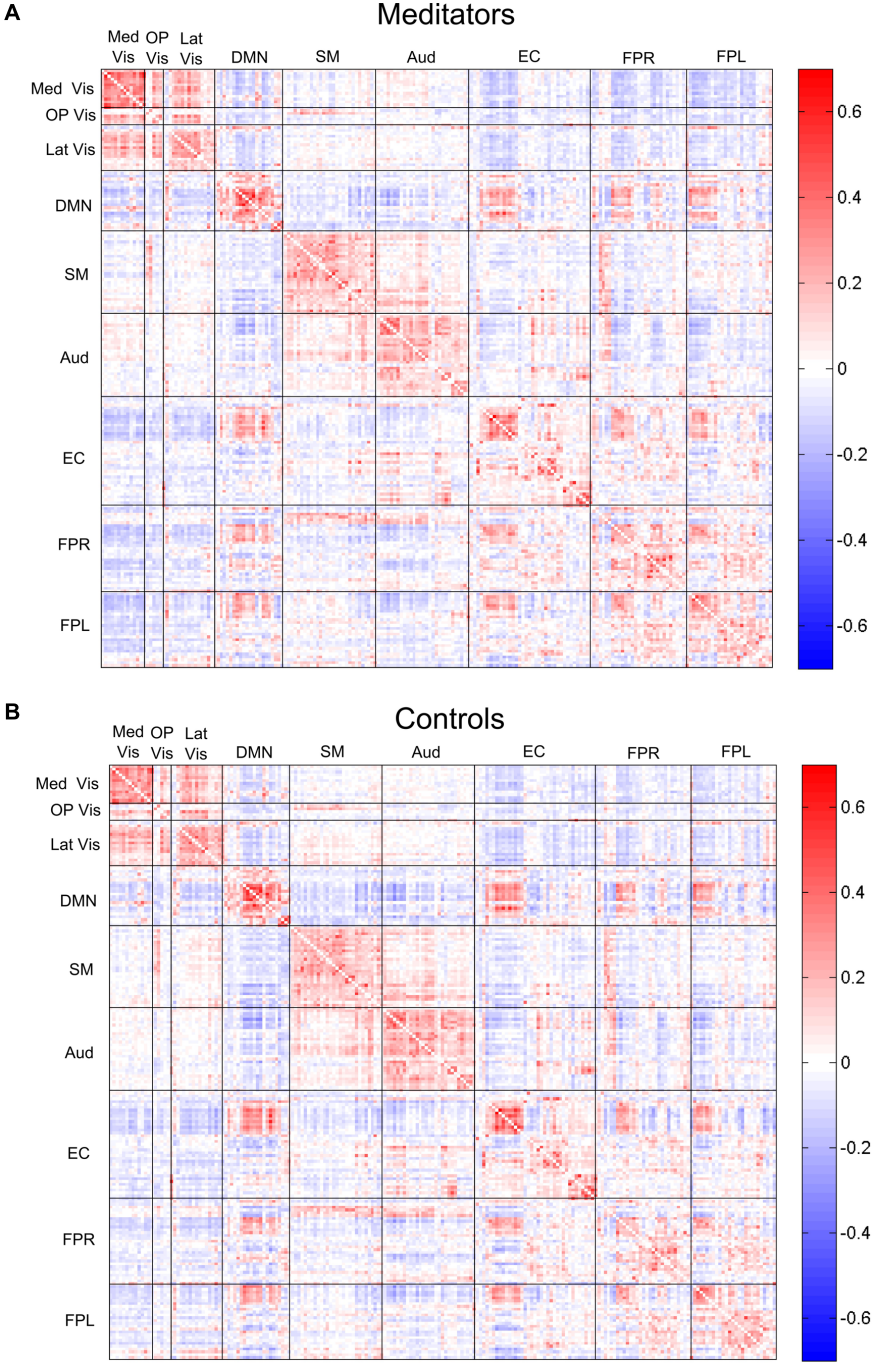
**Edgewise connectivity matrices, averaged by subject group.** The symmetric 209 × 209 connectivity matrices (as measured by Pearson correlations), averaged over the **(A)** meditators and **(B)** control groups. Edges are shown grouped by their module membership. Red edges indicate positive connectivity, while blue edges indicate negative connectivity.

The complete average correlation matrices, for meditators and controls, are shown in **Figure 4** as heat-maps, with edges grouped by their module membership. For the sake of interpretability, we separately examined the edges that were positive or negative in both groups average correlation maps, which represented the majority (82%) of the total edges.

**FIGURE 5:**
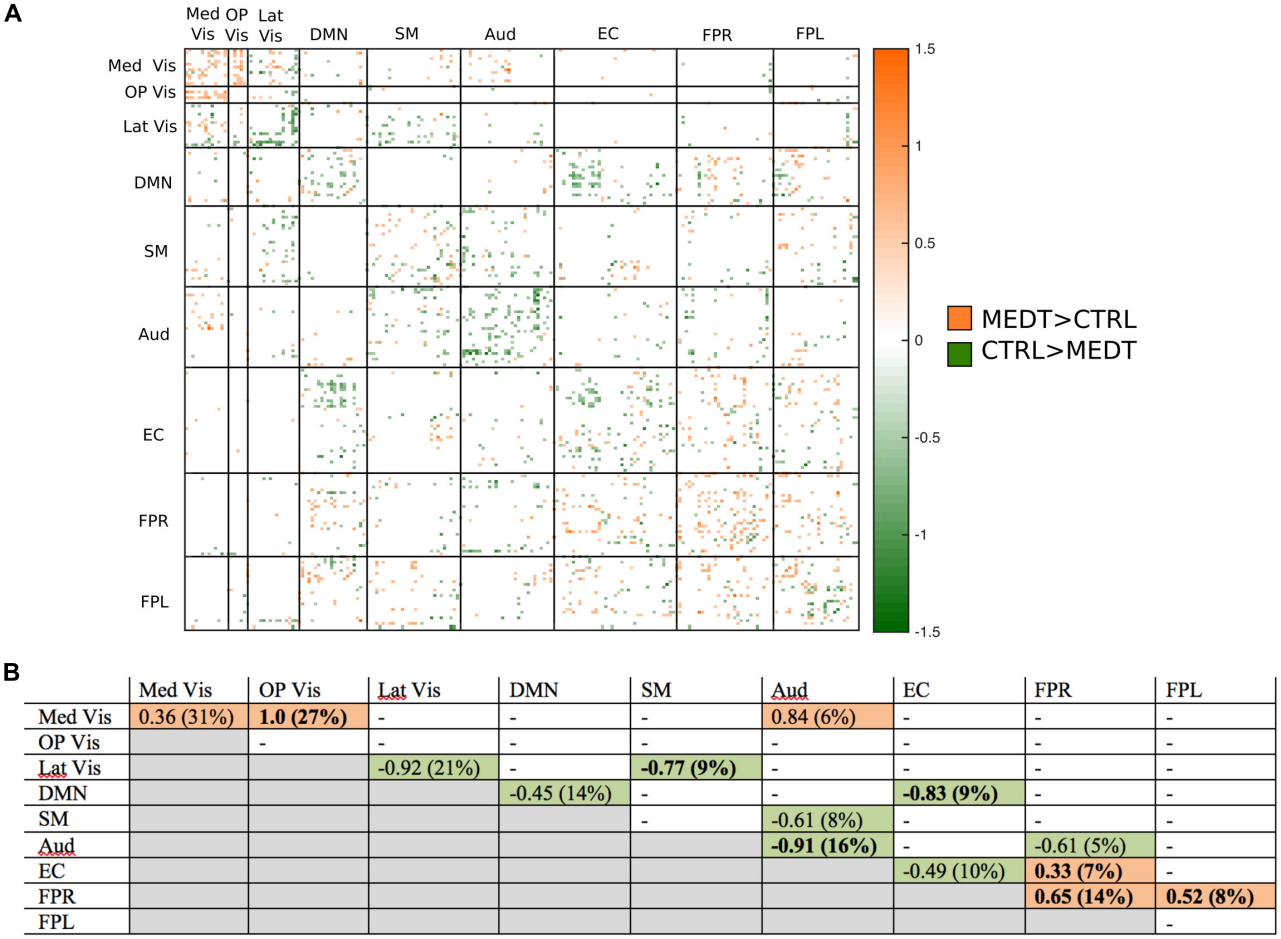
**Group differences in positive connectivity. (A)** For the edges that were *positive* on average, in both meditators and controls, we calculated the MEDT–CTRL standardized effect size to assess group differences; only edges with at least a moderate effect size (|*d*| > 0.5) are displayed and used in further analyses. **(B)** The results of the within-block consistency analysis are summarized as a table; for each module-to-module block containing at least 5% of edges meeting the required effect size threshold in **(A)**, the value of the consistency metric and the percent of edges retained post-thresholding (in parentheses) are reported. Shaded cells indicate blocks with a highly consistent edgewise effect, and are color-coded according to the direction of the group effect. Bolded values indicate blocks also highlighted in the modulewise analysis.

Of the 21,736 unique edgewise connections, 37% were positive in both groups, on average. **Figure 5A** displays positive edges with between-group difference of medium or larger effect size (i.e., |*d*| > 0.5; Cohen, 1988). In the figure, orange or green edges indicate that edgewise positive connectivity was higher in meditators or controls, respectively. For module-module blocks with at least 5% of edges exceeding the chosen effect size threshold, we computed the within-block consistency of the effect with the metric described in Section “Group Differences Based On Multivariate Pattern Analysis.” The value of the consistency metric, as well as the percent of the block’s edges retained post-thresholding, are displayed for each module-to-module block in **Figure 5B**. In this figure, shaded cells identify blocks with a highly consistent edgewise effect (i.e., consistency metric magnitude of at least 0.33); orange or green cells indicate that positive connections were higher among meditators or controls, respectively. Meditators had consistently higher positive connections than controls within the Med Vis and FPR modules, and for the Med Vis–OP Vis, Med Vis–Aud, EC–FPR, and FPR–FPL module pairs. Controls had higher positive connections than meditators within the Lat Vis, DMN, Aud, and EC modules, and for the following module pairs: Lat Vis–SM, DMN–EC, SM–Aud, and Aud–FPR. Module pairs that were also identified in the modulewise FC analysis (i.e., Med Vis–OP Vis, Lat Vis–SM, DMN–EC, Aud–Aud, EC–FPR, FPR–FPR, and FPR–FPL), are marked with bold font in **Figure 5B**.

**FIGURE 6:**
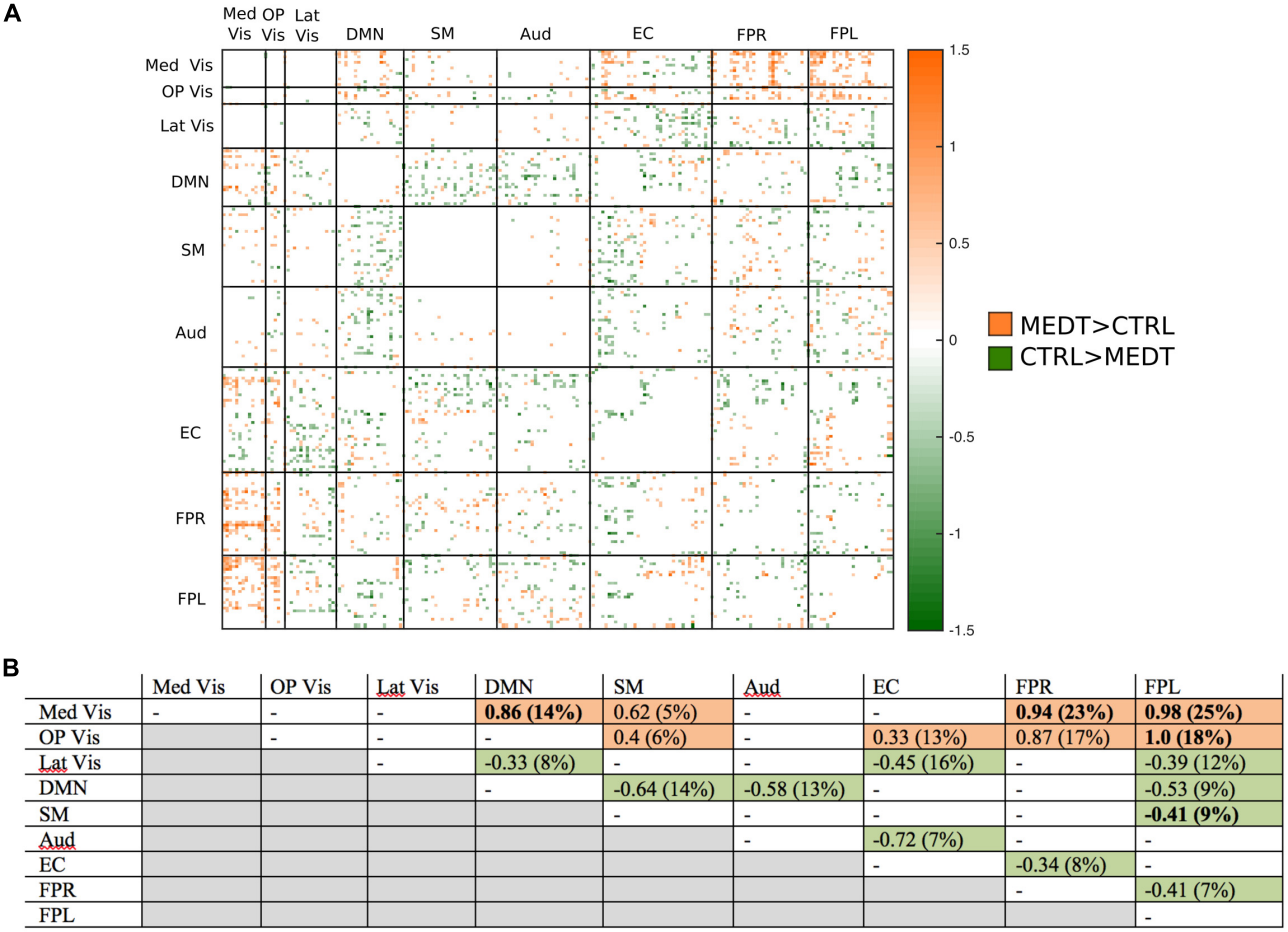
**Group differences in negative connectivity. (A)** For the edges that were negative on average, in both meditators and controls, we calculated the MEDT–CTRL standardized effect size to assess group differences; only edges with at least a moderate effect size (|*d*| > 0.5) are displayed and used in further analyses. **(B)** The results of the within-block consistency analysis are summarized as a table; for each module-to-module block containing at least 5% of edges meeting the required effect size threshold in **(A)**, the value of the consistency metric and the percent of edges retained post-thresholding (in parentheses) are reported. Shaded cells indicate blocks with a highly consistent edgewise effect, and are color-coded according to the direction of the group effect. Bolded values indicate blocks also highlighted in the modulewise analysis.

Of the 21,736 unique edgewise connections, 45% were negative in both groups, on average. **Figure 6A** displays negative edges with between-group difference of medium or larger effect size (i.e., |*d*| > 0.5; Cohen, 1988). In the figure, orange or green edges indicate that edgewise negative connectivity was higher in meditators or controls, respectively. With a similar approach to that used for positive connections, we identified module-module blocks with a highly consistent edgewise group difference in the negative connections and selected the blocks with at least 5% suprathreshold edges for further analysis. **Figure 6B** shows the value of the consistency metric and the percent of edges retained post-thresholding for each block. Shaded cells identify blocks with a highly consistent edgewise effect within the block, with orange, and green color corresponding to a modulewise negative connection stronger in meditators or controls, respectively. Meditators had consistently stronger negative connections than controls for the following module pairs: Med Vis–DMN, Med Vis–SM, Med Vis–FPR, Med Vis–FPL, OP Vis–SM, OP Vis–EC, OP Vis–FPR, OP Vis–FPL. Controls had consistently stronger negative connections than meditators for the Lat Vis–DMN, Lat Vis–EC, Lat Vis–FPL, DMN–SM, DMN–Aud, DMN–FPL, SM–FPL, Aud–EC, EC–FPR, and FPR–FPL module pairs. Module pairs that were also identified in the modulewise FC analysis (i.e., Med Vis–DMN, Med Vis–FPR, Med Vis–FPL, OP Vis–FPL, and SM–FPL), are marked with bold font in **Figure 6B**.

**FIGURE 7:**
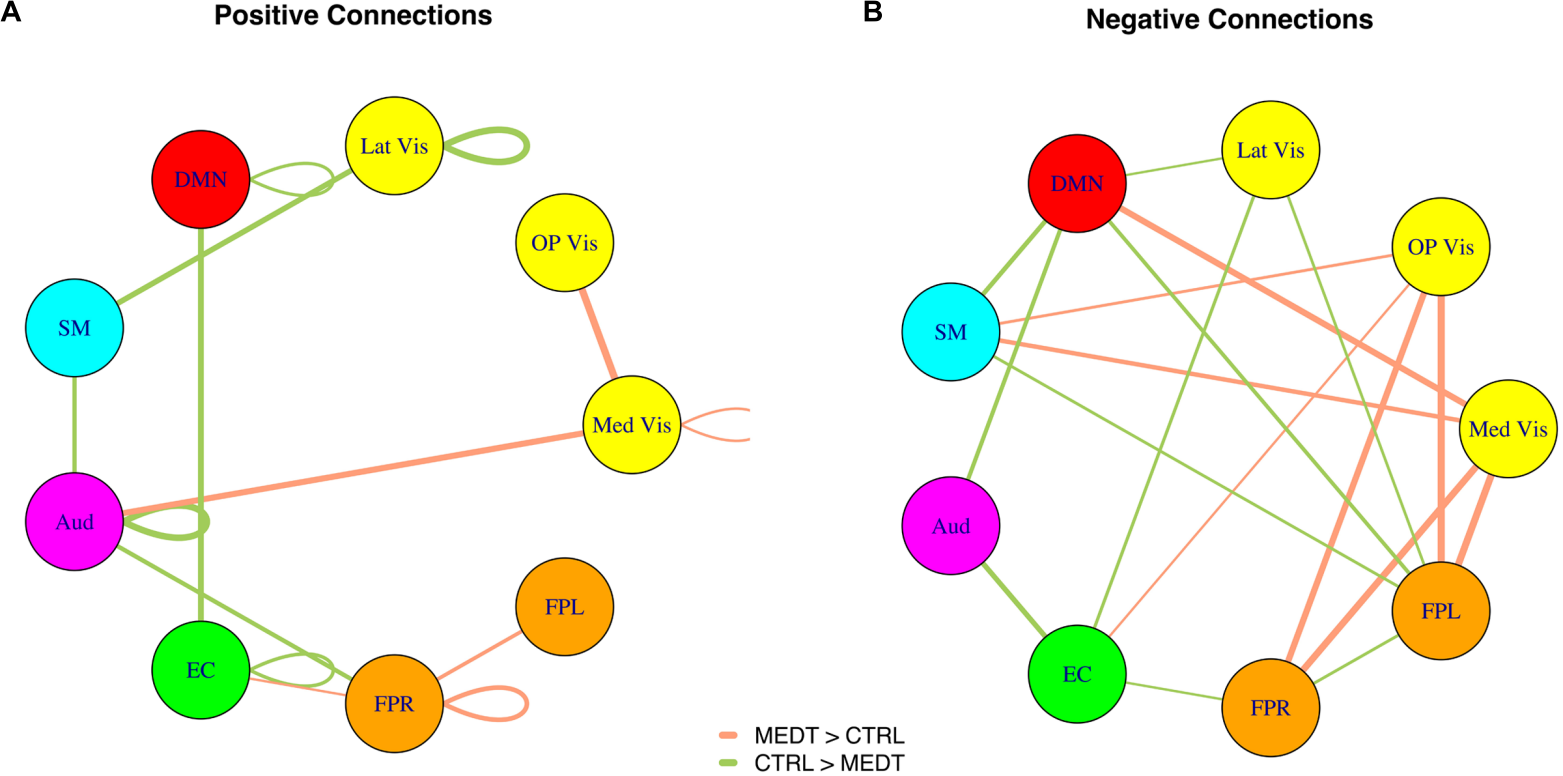
**Within-block consistency analysis results. (A)** Positive and **(B)** negative connections with a highly consistent group effect across the edges of a module-to-module block. Edge color indicates whether connections are stronger in meditators (orange) or controls (green), while the edge width reflects the value of the consistency metric.

Modulewise connections with consistent group effects for positive and negative connections are displayed in circular graphs in **Figure 7**, for easier comparison to the SVD-based results portrayed in **Figure 4**.

### Group Differences Based on Multivariate Pattern Analysis

**FIGURE 8:**
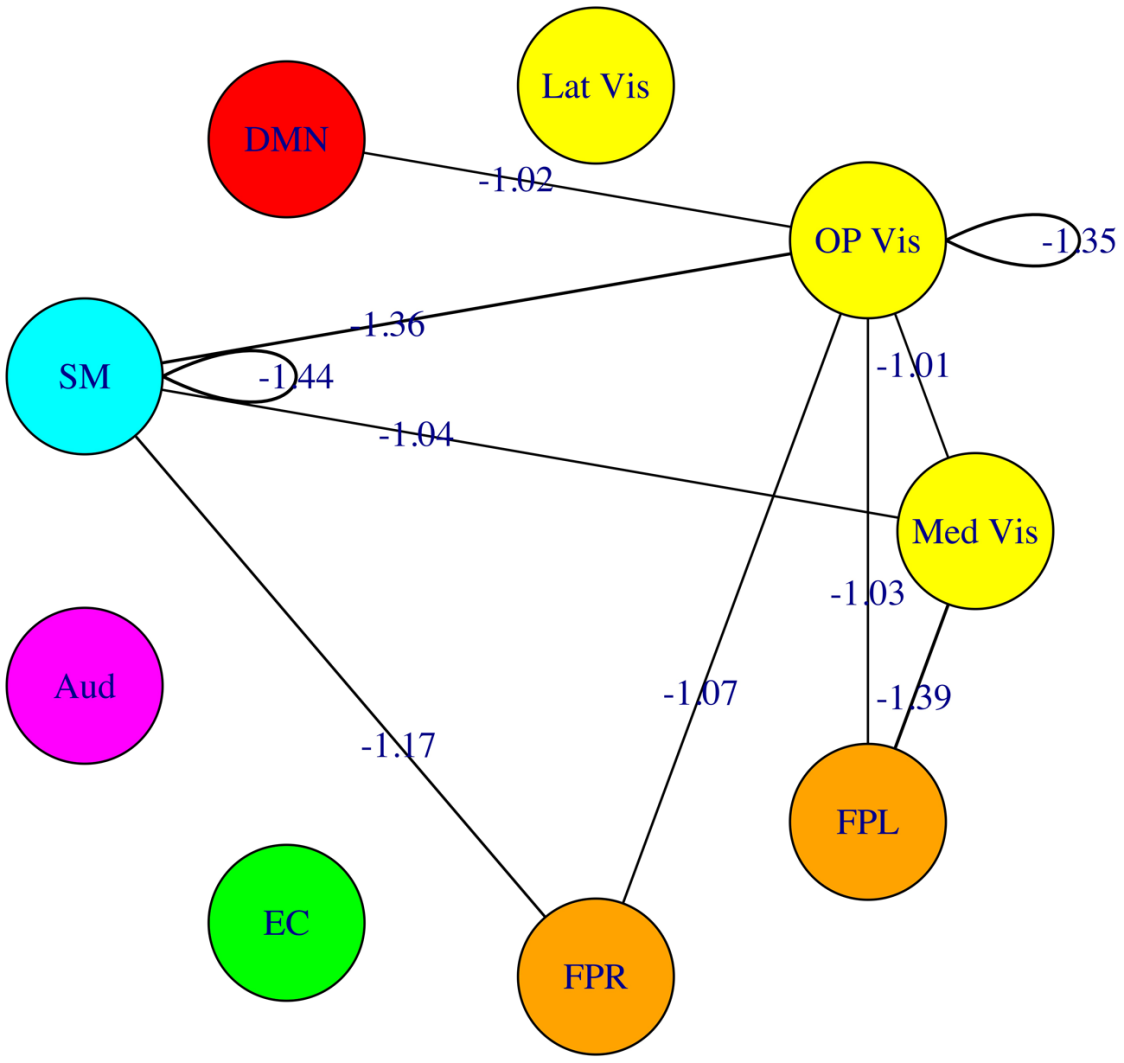
**Multivariate pattern analysis results.** The most heavily weighted modulewise-connectivities identified by the support vector machine (SVM) model, along with their connection weights. Considered jointly, these modulewise connections are the most critical for distinguishing the meditator and control groups.

The fourfold cross validation procedure for the connectivity-based SVM classifier using the 45 modulewise principal components yielded an overall accuracy rate of 79.2% (19/24 subjects correctly classified). The most heavily weighted modulewise connections in the SVM model, along with their weights, are displayed in **Figure 8**. They included the within OP Vis and SM module connections, and the following module–module connections: Med Vis–SM, Med Vis–OP Vis, Med Vis–FPL, OP Vis–FPL, OP Vis–FPR, OP Vis–SM, FPR–SM, and OP Vis–DMN. These connections, taken together, were the most critical for distinguishing meditators, and controls.

The fourfold cross-validation procedure for the second SVM analysis, aimed at classifying meditators and controls based on subjects’ CANTAB test scores yielded an overall accuracy rate of 75% (18/24 subjects correctly classified). The brain connectivity features demonstrated thus slightly better classification accuracy than the CANTAB scores. As an additional check, we also performed an SVM analysis using as input both the modulewise FC values (measured by first principal component coordinates) and the CANTAB scores: the model attained a classification rate of 79.2%, that is, the same rate achieved using the modulewise FC alone, showing that the amount of information embedded in the brain FC measures, for what concerns subjects’ classification, was not significantly augmented by further inclusion of the employed neuropsychological tests’ scores.

**FIGURE 9:**
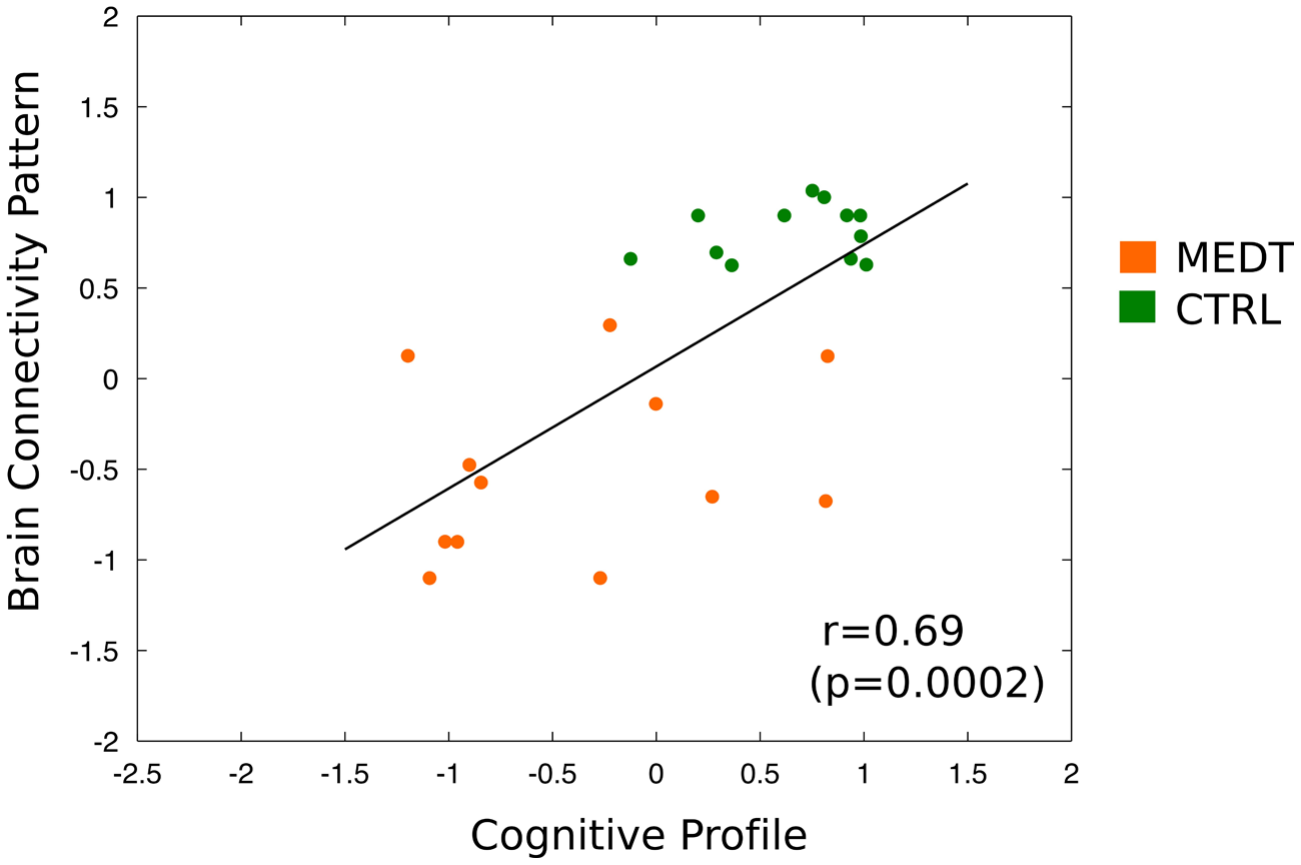
**Correlation of brain-based and neuropsychology-based SVM classification scores across all subjects.** Scatterplot and linear fitting of dimensional classification scores from the SVM using the CANTAB test scores and from the SVM using the first PC of modulewise brain connectivity. The two sets of scores were strongly correlated across subjects (*r* = 0.69, *p* = 0.0002), indicating that subjects classified as meditators based on their pattern of brain connectivity, also tended to be classified as meditators based on their performance on the neuropsychological tests.

Finally, the dimensional classification scores from the connectivity- and CANTAB-based SVM were strongly correlated (*r* = 0.69, *p* = 0.002; see **Figure 9**); demonstrating that subjects with more “meditator-like” patterns of connectivity also exhibited more “meditator-like” patterns of CANTAB test performance.

### Association between Brain Connectivity and Neuropsychological Tests

The correlation analysis across all subjects (MEDT + CTRL) of the 45 modulewise functional connectivity strengths (summarized by the first principal component coordinates extracted from SVD) and attentional performance, as indexed by RVIP test scores, identified several associations with a large or greater effect size (i.e., |*r*| > 0.5; Cohen, 1988), reported in **Table 2**. The OP Vis–EC, OP Vis–FPR, DMN–FPR, EC–FPR, and FPR–FPL connections were associated with RVIP target sensitivity, while the OP Vis–EC, OP Vis–FPR, OP Vis–FPL, SM–EC, and Aud–FPR connections were associated with RVIP reaction time.

**Table 2:** The correlation of rapid visual information processing (RVIP) test performance with the 45 modulewise first principal component coordinates revealed these large associations.

	Modulewise connections	Correlation with RVIP task	*p*-value (uncorrected)
RVIP sensitivity to target	OP Vis – EC OP Vis – FPR DMN – FPR EC – FPR FPR – FPL	0.54 -0.53 0.51 0.56 0.60	0.006 0.008 0.010 0.004 0.002

RVIP response time	OP Vis – EC OP Vis – FPR OP Vis – FPL SM – EC Aud – FPR	-0.61 0.51 0.51 0.51 -0.59	0.002 0.011 0.010 0.010 0.003

## Discussion

Network analyses of fMRI data are increasingly being used to characterize patterns of brain dynamics associated with a specific mental state, trait, or clinical condition (Filippi et al., 2013; Smith et al., 2013). It has also been recently shown that MRI-based functional connectivity strength is spatially well-matched to the local distribution of regional cerebral blood flow (rCBF), and thus is likely to reflect the degree of neural activation (Liang et al., 2013). While previous studies have employed functional connectivity for the investigation of the neural correlates of contemplative practices (Farb et al., 2007; Josipovic et al., 2011; Kilpatrick et al., 2011; Hasenkamp and Barsalou, 2012; Lehmann et al., 2012; Pagnoni, 2012; Taylor et al., 2013; Garrison et al., 2014; Marzetti et al., 2014), the application of specific techniques from graph theory to this field of research is still scarce (see Gard et al., 2014, for an exception). In the present study, we examined how a group of habitual Zen practitioners and a group of matched control subjects differed on selected MRI-based functional connectivity network measures during a simplified (attention-to-breathing) meditative task without any external experimental stimulation. We also explored the relationship of these measures with the performance of the same subjects on a computerized test of sustained attention (RVIP).

A first, summary outlook on the group differences in functional connectivity is provided by the modulewise (SVD-based) analysis. This analysis revealed a differential group pattern where meditators were characterized by frontoparietal attentional circuits (FPR, FPL) having stronger positive connections to an anterior cingulate-insula-caudate network (EC) involved in executive processing and saliency detection, and stronger negative connections to early visual areas (OP Vis, Med Vis), compared to controls; also, meditators exhibited on average weaker positive connections between DMN and the saliency network (EC), and stronger negative connection between DMN and early visual areas (OP Vis, Med Vis).

A complementary analysis, examining the consistency of the edgewise connection strengths within each module-module block, largely confirmed the above findings but also revealed a more nuanced picture, with additional group connectivity differences involving, among others, a weaker negative connectivity, for meditators compared to controls, between the DMN and both the auditory (Aud) and the sensorimotor (SM) networks. Finally, a direct assessment of the subspace of modulewise connectivity that could best separate meditators from control subjects, via an SVM classifier, underscored again the importance of connections between frontoparietal circuits (FPR, FPL) and early visual areas (Med Vis, OP Vis), but also between these occipital regions and both sensorimotor cortex (SM) and DMN.

These results are meaningful, as frontoparietal circuits and the DMN have long been assumed to be affected by contemplative practice. Meditation generally involves a regulation of attentional processes and spontaneous mentation or mind-wandering, along with an increase in meta-awareness (awareness of one’s own mental processes). Attentional deployment and regulation are known to impinge crucially on frontoparietal circuits (Corbetta and Shulman, 2002), while DMN has been consistently implicated in mind-wandering, both in and outside the context of meditation (Binder et al., 1999; Mason et al., 2007; Christoff et al., 2009; Brewer et al., 2011). The stronger positive connection between frontoparietal circuits and the saliency network exhibited by meditators in the present study, may be linked the vigilant attitude that meditators aim to keep in order to detect and become aware of the fluctuations in one’s own mental state, so that salient events in the mental landscape are accompanied by an activation of regulatory attentional mechanisms. Interestingly, the saliency network has been shown to activate during awareness of episodes of mind-wandering in meditation, an effect putatively ascribed to the fact that mind-wandering episodes represent a violation of the target of remaining concentrated on breathing and thus may trigger neural activity in conflict-processing and arousal-related areas such as anterior cingulate and insular cortices (Hasenkamp et al., 2012). In this perspective, the observed weaker positive connection between the EC and the DMN modules, for meditators compared to controls, could reflect the less-judgmental and more accepting attitude of meditators vis-à-vis the spontaneous occurrence of mind-wandering episodes, compared to control subjects who, because of their lack of experience, may in fact react more emotionally to such violations of the concentrative goal of the task.

Meditators also exhibited weaker *negative* connections between the DMN and a number of modules that process sensory and motor information (Aud, Lat Vis, SM; see **Figure 7B**), one possible interpretation is that, in controls, the spontaneous activation of DMN during episodes of mind-wandering corresponds to a more active dampening, compared to meditators, of the information from channels linked to the external environment, leading to sensorimotor decoupling (Kam and Handy, 2013), and thus to increased mental absorption in internally generated, distracting thoughts. (The stronger negative connection in meditators between the DMN and medial visual cortex would represent, however, an exception to this general pattern).

Of particular interest is the DMN within-module positive connectivity, which was weaker in meditators compared to controls (**Figure 7A**). If this can be taken to reflect a less active DMN in meditators during attention-to-breathing, it would be in good agreement with the finding of a similar effect based on an independent measure of the frequency of activation (the skewness of the BOLD distribution) of the main node of the DMN, the retrosplenial cortex, that we had previously observed in the same data (Pagnoni, 2012). The possibility that meditation could provide intervals of momentary respite for an overactive DMN and perhaps, through regular practice, even induce a lasting regularization of the activity in the same circuit outside of formal practice, is especially intriguing. Recent evidence has linked the process of deposition of beta-amyloid peptide and brain atrophy in Alzheimer’s disease (AD) – a process whose spatial pattern strikingly resembles the DMN’s layout (Buckner et al., 2005) – to the sustained metabolic activity of the DMN (Walker and Jucker, 2011). Although more research is obviously needed, the prospect that the regular practice of certain kind of contemplative techniques could prove even mildly protective against the onset of AD, should not be neglected.

Meditators also exhibited stronger within-module positive connectivity in the FPR (**Figures 3** and **7A**) – a network known for its relevance in sustained attention (Coull et al., 1996; Sarter et al., 2001; Lim et al., 2010) – suggesting an increased engagement of the latter during attention-to-breathing in meditators by virtue of their practice. Enhanced activation of the FPR network has been previously associated with better performance in the RVIP task (Lawrence et al., 2003); such a finding, together with the better performance of this sample of meditators in the RVIP task when controlling for age (Pagnoni and Cekic, 2007) and the prominent featuring of the FPR module in the pool of important correlations of brain connectivity strengths with RVIP performance (**Table 2**), lends further support to the notion of an increased engagement of attentional processes in meditators, compared to controls, during the attention-to-breathing meditative task. Of note, frontoparietal circuits (FPR, FPL) were also more negatively connected to early visual areas (Med Vis, OP Vis) in meditators compared to controls (**Figures 3** and **7B**), a possible correlate of a more effective redeployment of attention toward internally generated stimuli in meditators (e.g., respiratory sensations, monitoring of the appearance of spontaneous thoughts) during the attention-to-breathing task (Cooper et al., 2003). The hypothesis that the strength of the negative connections between frontoparietal and early visual areas during a task that prescribes *inward* attention is related to the individual ability to voluntarily regulate attention may be supported by the finding that subjects who exhibited a stronger negative correlation between FPR/FPL and OP Vis also displayed a better performance in the RVIP sustained attention task (**Table 2**).

An important result is that the module-module connection strengths were able to predict the subjects’ group membership via the SVM classifier with quite a high accuracy (79%). A similar SVM analysis based on CANTAB cognitive test scores also produced a reasonable (but inferior) classification rate (75%). The SVM also provided a further characterization of the distinction between meditators and control subjects, via the dimensional classification scores. Correlation analyses showed that the dimensional classification scores from the connectivity-based SVM were significantly associated with the dimensional classification scores from the CANTAB-based SVM, indicating that subjects with more “meditator-like” patterns of connectivity also demonstrated more “meditator-like” patterns of performance in the employed set of neuropsychological tests.

### Limitations and Future Directions

We examined the effects of meditation on rsFC across the whole brain using a multi-stage network approach. Our study included a well-controlled matched sample of habitual meditators and meditation-naïve controls during a basic attention-to-breathing fMRI protocol. The limited sample size (*n*_MEDT_ = 12, *n*_CTRL_ = 12), along with the large number of examined nodes, and connections, made straightforward multiple comparisons correction of the *p*-values impractical (and overly conservative, given the non-independence of the nodes). Since the effect size, on the other hand, is not confounded by the sample size (Lang et al., 1998), we chose to report only the findings with a medium or larger effect size (along with uncorrected *p*-values), acknowledging the fact that such findings should be regarded as having principally an exploratory, hypothesis-generating role.

Also, in our analysis, we calculated the Pearson’s correlation coefficients between the time series of two nodes to represent their functional connectivity strength. Correlation is one of the simplest and most commonly used association measures, but it does not imply direct connections between two nodes (i.e., a third node may be mediating their relationship). Partial correlation, which regresses out confounding nodes to distinguish direct from indirect connections, can better estimate the true connectivity network. In fact, some differences between the univariate and multivariate results portrayed in **Figures 3** and **8**, respectively, could probably be reduced, were partial correlations to be employed. Partial correlations can be estimated via the inverse of the covariance matrix, but the algorithm presents computational difficulties when the number of columns of the correlation matrix exceeds the number of rows that we were not able to resolve at the time of writing. Smith et al. (2011) showed that partial correlation performs better than regular correlation in a variety of simulations of fMRI data, and we plan to adopt this approach in future analyses.

Finally, the reported findings indicate that the long-term practice of meditation may be associated with FC changes in several RSNs. However, as this is not a randomized study, the causal direction of the observed association cannot be determined. A longitudinal study, despite a number of issues that make it practically very problematic when aimed at studying the effects of long term training (e.g., subjects’ compliance, study dropout, intervening confounding variables), would be necessary to establish causality in a decisive way.

## Conflict of Interest Statement

The authors declare that the research was conducted in the absence of any commercial or financial relationships that could be construed as a potential conflict of interest.

## References

[B1] Allen E. A. Erhardt E. B. Damaraju E. Gruner W. Segall J. M. Silva R. F. (2011). A baseline for the multivariate comparison of resting state networks. *Front. Neurosci.* 5:2 10.3389/fnsys.2011.00002 PMC3051178 21442040

[B2] Bassett D. S. Bullmore E. Verchinski B. A. Mattay V. S. Weinberger D. R. Meyer-Lindenberg A. (2008). Hierarchical organization of human cortical networks in health and schizophrenia. *J. Neurosci.* 28 9239–9248 10.1523/JNEUROSCI.1929-08.2008 18784304 PMC2878961

[B3] Binder J. R. Frost J. A. Hammeke T. A. Bellgowan P. S. Rao S. M. Cox R. W. (1999). Conceptual processing during the conscious resting state. A functional MRI study. *J Cogn. Neurosci* 11 80–95 10.1162/089892999563265 9950716

[B4] Biswal B. Yetkin R. Z. Haughton V. M. Hyde J. S. (1995). Functional connectivity in the motor cortex of resting human brain using echo-planar MRI. *Magn. Reson. Med.* 34 537–541 10.1002/mrm.1910340409 8524021

[B5] Brewer J. A. Worhunsky P. D. Gray J. R. Tang Y. Y. Weber J. Kober H. (2011). Meditation experience is associated with differences in default mode network activity and connectivity. *Proc. Natl. Acad. Sci. U.S.A.* 108 20254–20259 10.1073/pnas.1112029108 22114193 PMC3250176

[B6] Buckner R. L. Snyder A. Z. Shannon B. J. LaRossa G. Sachs R. Fotenos A. F. (2005). Molecular, structural, and functional characterization of Alzheimer’s disease: evidence for a relationship between default activity, amyloid, and memory. *J. Neurosci.* 25 7709–7717 10.1523/JNEUROSCI.2177-05.2005 16120771 PMC6725245

[B7] Chang C. C. Lin C. J. (2011). LIBSVM: a library for support vector machines. *ACM Trans. Intell. Sys. Technol.* (*TIST*) 2 27 10.1145/1961189.1961199

[B8] Christoff K. Gordon A. M. Smallwood J. Smith R. Schooler J. W. (2009). Experience sampling during fMRI reveals default network and executive system contributions to mind wandering. *Proc. Natl. Acad. Sci. U.S.A.* 106 8719–8724 10.1073/pnas.0900234106 19433790 PMC2689035

[B9] Cohen J. (1988). *Statistical Power Analysis for the Behavioral Sciences* 2nd Edn. Hillsdale, MI: Lawrence Erlbaum.

[B10] Cooper N. R. Croft R. J. Dominey S. J. J. Burgess A. P. Gruzelier J. H. (2003). Paradox lost? Exploring the role of alpha oscillations during externally vs. internally directed attention and the implications for idling and inhibition hypotheses. *Int. J. Psychophysiol.* 47 65–74 10.1016/S0167-8760(02)00107-1 12543447

[B11] Corbetta M. Shulman G. L. (2002). Control of goal-directed and stimulus-driven attention in the brain. *Nat. Rev. Neurosci.* 3 201–215 10.1038/nrn755 11994752

[B12] Coull J. T. Frith C. D. Frackowiak R. S. Grasby P. M. (1996). A fronto-parietal network for rapid visual information processing: a PET study of sustained attention and working memory. *Neuropsychologia* 34 1085–1095 10.1016/0028-3932(96)00029-2 8904746

[B13] Csardi G. Nepusz T. (2006). The igraph software package for complex network research. *Inter. J. Complex Syst.* 1695 1–9.

[B14] Dosenbach N. U. F. Nardos B. Cohen A. L. Fair D. A. Power J. D. Church J. A. (2010). Prediction of individual brain maturity using fMRI. *Science* 329 1358–1361 10.1126/science.1194144 20829489 PMC3135376

[B15] Farb N. A. S. Segal Z. V. Anderson A. K. (2013). Mindfulness meditation training alters cortical representations of interoceptive attention. *Soc. Cogn. Affect. Neurosci.* 8 15–26 10.1093/scan/nss066 22689216 PMC3541492

[B16] Farb N. A. S. Segal Z. V. Mayberg H. Bean J. McKeon D. Fatima Z. (2007). Attending to the present: mindfulness meditation reveals distinct neural modes of self-reference. *Soc. Cogn. Affect. Neurosci.* 2 313–322 10.1093/scan/nsm030 18985137 PMC2566754

[B17] Filippi M. van den Heuvel M. P. Fornito A. He Y. Hulshoff Pol H. E. Agosta F. (2013). Assessment of system dysfunction in the brain through MRI-based connectomics. *Lancet Neurol.* 12 1189–1199 10.1016/S1474-4422(13)70144-3 24120645

[B18] Fornito A. Zalesky A. Bullmore E. T. (2010). Network scaling effects in graph analytic studies of human resting-state fMRI data. *Front. Syst. Neurosci.* 4:22 10.3389/fnsys.2010.00022 PMC2893703 20592949

[B19] Fox M. D. Raichle M. E. (2007). Spontaneous fluctuations in brain activity observed with functional magnetic resonance imaging. *Nat. Rev. Neurosci.* 8 700–711 10.1038/nrn2201 17704812

[B20] Fransson P. (2005). Spontaneous low-frequency BOLD signal fluctuations: an fMRI investigation of the resting-state default mode of brain function hypothesis. *Hum. Brain Mapp.* 26 15–29 10.1002/hbm.20113 15852468 PMC6871700

[B21] Froeliger B. Garland E. L. Kozink R. V. Modlin L. A. Chen N. K. McClernon F. J. (2012). Meditation-state functional connectivity (msFC): strengthening of the dorsal attention network and beyond. *Evid. Based Complement. Alternat. Med.* 2012:680407 10.1155/2012/680407 PMC3320106 22536289

[B22] Gard T. Taquet M. Dixit R. Hölzel B. K. de Montjoye Y. A. Brach N. (2014). Fluid intelligence and brain functional organization in aging yoga and meditation practitioners. *Front. Aging Neurosci.* 6:76 10.3389/fnagi.2014.00076 PMC4001007 24795629

[B23] Garrison K. A. Scheinost D. Constable R. T. Brewer J. A. (2014). BOLD signal and functional connectivity associated with loving kindness meditation. *Brain Behav.* 4 337–347 10.1002/brb3.219 24944863 PMC4055184

[B24] Greicius M. D. Srivastava G. Reiss A. L. Menon V. (2004). Default-mode network activity distinguishes Alzheimer’s disease from healthy aging: evidence from functional MRI. *Proc. Natl. Acad. Sci. U.S.A.* 101 4637–4642 10.1073/pnas.0308627101 15070770 PMC384799

[B25] Guo Y. (2011). A general probabilistic model for group independent component analysis and its estimation methods. *Biometrics* 67 1532–1542 10.1111/j.1541-0420.2011.01601.x 21517789 PMC3412593

[B26] Guo Y. Pagnoni G. (2008). A unified framework for group independent component analysis for multi-subject fMRI data. *Neuroimage* 42 1078–1093 10.1016/j.neuroimage.2008.05.008 18650105 PMC2853771

[B27] Guo Y. Tang L. (2013). A hierarchical model for probabilistic independent component analysis of multi-subject fMRI studies. *Biometrics* 69 970–981 10.1111/biom.12068 24033125 PMC4130464

[B28] Hasenkamp W. Barsalou L. W. (2012). Effects of meditation experience on functional connectivity of distributed brain networks. *Front. Hum. Neurosci.* 6:38 10.3389/fnhum.2012.00038 PMC3290768 22403536

[B29] Hasenkamp W. Wilson-Mendenhall C. D. Duncan E. Barsalou L. W. (2012). Mind wandering and attention during focused meditation: a fine-grained temporal analysis of fluctuating cognitive states. *Neuroimage* 59 750–760 10.1016/j.neuroimage.2011.07.008 21782031

[B30] Jang J. H. Jung W. H. Kang D. H. Byun M. S. Kwon S. J. Choi C. H. (2011). Increased default mode network connectivity associated with meditation. *Neurosci. Lett.* 487 358–362 10.1016/j.neulet.2010.10.056 21034792

[B31] Josipovic Z. Dinstein I. Weber J. Heeger D. J. (2011). Influence of meditation on anti-correlated networks in the brain. *Front. Hum. Neurosci.* 5:183 10.3389/fnhum.2011.00183 PMC3250078 22287947

[B32] Kam J. W. Y. Handy T. C. (2013). The neurocognitive consequences of the wandering mind: a mechanistic account of sensory-motor decoupling. *Front. Psychol.* 4:725 10.3389/fpsyg.2013.00725 PMC3796327 24133472

[B33] Kilpatrick L. A. Suyenobu B. Y. Smith S. R. Bueller J. A. Goodman T. Creswell J. D. (2011). Impact of mindfulness-based stress reduction training on intrinsic brain connectivity. *Neuroimage* 56 290–298 10.1016/j.neuroimage.2011.02.034 21334442 PMC3072791

[B34] Laird A. R. Fox P. M. Eickhoff S. B. Turner J. A. Ray K. L. McKay D. R. (2011). Behavioral interpretations of intrinsic connectivity networks. *J. Cogn. Neurosci.* 23 4022–4037 10.1162/jocn_a_00077 21671731 PMC3690655

[B35] Lang J. M. Rothman K. J. Cann C. I. (1998). That confounded p-value. *Epidemiology* 9 7–8 10.1097/00001648-199801000-00004 9430261

[B36] Lawrence N. Ross T. Hoffmann R. Garavan H. Stein E. (2003). Multiple neuronal networks mediate sustained attention. *J. Cogn. Neuroscie.* 15 1028–1038 10.1162/089892903770007416 14614813

[B37] Lehmann D. Faber P. L. Tei S. Pascual-Marqui R. D. Milz P. Kochi K. (2012). Reduced functional connectivity between cortical sources in five meditation traditions detected with lagged coherence using EEG tomography. *Neuroimage* 60 1574–1586 10.1016/j.neuroimage.2012.01.042 22266174

[B38] Liang X. Zou Q. He Y. Yang Y. (2013). Coupling of functional connectivity and regional cerebral blood flow reveals a physiological basis for network hubs of the human brain. *Proc. Natl. Acad. Sci. U.S.A.* 110 1929–1934 10.1073/pnas.1214900110 23319644 PMC3562840

[B39] Lim J. Wu W. C. Wang J. Detre J. A. Dinges D. F. Rao H. (2010). Imaging brain fatigue from sustained mental workload: an ASL perfusion study of the time-on-task effect. *Neuroimage* 49 3426–3435 10.1016/j.neuroimage.2009.11.020 19925871 PMC2830749

[B40] Lowe M. J. (2012). The emergence of doing “nothing” as a viable paradigm design. *Neuroimage* 62 1146–1151 10.1016/j.neuroimage.2012.01.014 22245648

[B41] Marzetti L. Di Lanzo C. Zappasodi F. Chella F. Raffone A. Pizzella V. (2014). Magnetoencephalographic alpha band connectivity reveals differential default mode network interactions during focused attention and open monitoring meditation. *Front. Hum. Neurosci.* 8:832 10.3389/fnhum.2014.00832 PMC4197643 25360102

[B42] Mason M. F. Norton M. I. Horn J. D. V. Wegner D. M. Grafton S. T. Macrae C. N. (2007). Wandering minds: the default network and stimulus-independent thought. *Science* 315 393–395 10.1126/science.1131295 17234951 PMC1821121

[B43] Pagnoni G. (2012). Dynamical properties of BOLD activity from the ventral posteromedial cortex associated with meditation and attentional skills. *J. Neurosci.* 32 5242–5249 10.1523/JNEUROSCI.4135-11.2012 22496570 PMC3362741

[B44] Pagnoni G. Cekic M. (2007). Age effects on gray matter volume and attentional performance in Zen meditation. *Neurobiol. Aging* 28 1623–1627 10.1016/j.neurobiolaging.2007.06.008 17655980

[B45] Pagnoni G. Cekic M. Guo Y. (2008). “Thinking about not-thinking”: neural correlates of conceptual processing during Zen meditation. *PLoS ONE* 3:e3083 10.1371/journal.pone.0003083 PMC2518618 18769538

[B46] Power J. D. Cohen A. L. Nelson S. M. Wig G. S. Barnes K. A. Church J. A. (2011). Functional network organization of the human brain. *Neuron* 72 665–678 10.1016/j.neuron.2011.09.006 22099467 PMC3222858

[B47] Raichle M. E. MacLeod A. M. Snyder A. Z. Powers W. J. Gusnard D. A. Shulman G. L. (2001). A default mode of brain function. *Proc. Natl. Acad. Sci. U.S.A.* 98 676–682 10.1073/pnas.98.2.676 11209064 PMC14647

[B48] Sahakian B. J. Owen A. M. (1992). Computerized assessment in neuropsychiatry using CANTAB: discussion paper. *J. R. Soc. Med.* 85 399–402. 1629849 PMC1293547

[B49] Sarter M. Givens B. Bruno J. P. (2001). The cognitive neuroscience of sustained attention: where top-down meets bottom-up. *Brain Res. Rev.* 35 146–160 10.1016/S0165-0173(01)00044-3 11336780

[B50] Satterthwaite T. D. Wolf D. H. Roalf D. R. Ruparel K. Erus G. Vandekar S. (2014). Linked sex differences in cognition and functional connectivity in youth. *Cereb. Cortex* 10.1093/cercor/bhu036 [Epub ahead of print]. PMC4537416 24646613

[B51] Smith S. M. Fox P. T. Miller K. L. Glahn D. C. Fox P. M. Mackay C. E. (2009). Correspondence of the brain’s functional architecture during activation and rest. *Proc. Natl. Acad. Sci. U.S.A.* 106 13040–13045 10.1073/pnas.0905267106 19620724 PMC2722273

[B52] Smith S. M. Miller K. L. Salimi-Khorshidi G. Webster M. Beckmann C. F. Nichols T. E. (2011). Network modelling methods for FMRI. *Neuroimage* 54 875–891 10.1016/j.neuroimage.2010.08.063 20817103

[B53] Smith S. M. Vidaurre D. Beckmann C. F. Glasser M. F. Jenkinson M. Miller K. L. (2013). Functional connectomics from resting-state fMRI. *Trends Cogn. Sci.* 17 666–682 10.1016/j.tics.2013.09.016 24238796 PMC4004765

[B54] Taylor V. A. Daneault V. Grant J. Scavone G. Breton E. Roffe-Vidal S. (2013). Impact of meditation training on the default mode network during a restful state. *Soc. Cogn. Affect. Neurosci.* 8 4–14 10.1093/scan/nsr087 22446298 PMC3541485

[B55] Tzourio-Mazoyer N. Landeau B. Papathanassiou D. Crivello F. Etard O. Delcroix N. (2002). Automated anatomical labeling of activations in SPM using a macroscopic anatomical parcellation of the MNI MRI single-subject brain. *Neuroimage* 15 273–289 10.1006/nimg.2001.0978 11771995

[B56] Walker L. C. Jucker M. (2011). Amyloid by default. *Nat. Neurosci.* 14 669–670 10.1038/nn.2853 21613991 PMC10715806

[B57] Xia M. Wang J. He Y. (2013). BrainNet Viewer: a network visualization tool for human brain connectomics. *PLoS ONE* 8:e68910 10.1371/journal.pone.0068910 PMC3701683 23861951

[B58] Zuo X. N. Kelly C. Adelstein J. S. Klein D. F. Castellanos F. X. Milham M. P. (2010). Reliable intrinsic connectivity networks: test–retest evaluation using ICA and dual regression approach. *Neuroimage* 49 2163–2177 10.1016/j.neuroimage.2009.10.080 19896537 PMC2877508

